# Impact of tearing spermatic cords during castration in live and dead piglets and consequences on welfare

**DOI:** 10.1186/s40813-021-00200-7

**Published:** 2021-02-14

**Authors:** Simone M. Schmid, Chiara I. Genter, Céline Heinemann, Julia Steinhoff-Wagner

**Affiliations:** Institute of Animal Science, University of Bonn, 53115 Bonn, Germany

**Keywords:** Animal health, Animal welfare, Castration management, Mutilation, Pain assessment, Pain behavior, Pork production

## Abstract

**Background:**

Although the tearing of tissues during castration is forbidden in the EU, it is still routinely applied in many countries. The goal of this study was to evaluate vocalizations and movements of male piglets undergoing castration by applying different techniques and pain treatments based on scores under practical conditions (Trial 1, *n* = 50) and to investigate anatomical features after castration of dead piglets with different techniques (Trial 2, *n* = 28).

**Results:**

In Trial 1, treatment groups did not significantly influence the duration of castration. Both the duration of vocalization and the scores for vocalizations and movements were lower in piglets castrated under general anesthesia (*P* < 0.05). Behavior scores in conscious piglets did not differ. The incision and extraction caused lower vocalizations and movements than the pulling and severing of spermatic cords (*P* < 0.01). Movements were more intense during tearing of the spermatic cords than during cutting at the first and second severing (*P* < 0.01). In both trials, the remains of spermatic cords protruded tendentially more often from castration wounds after severing by tearing (*P* < 0.09). In Trial 2, the minimum, mean and maximum lengths of the testicles and spermatic cords were extended when severing was realized by tearing (*P* < 0.01). The mean relative testicle weight of 1.05‰ in dead piglets castrated by tearing was larger than that in dead piglets castrated by cutting (0.91‰) (*P* < 0.05).

**Conclusions:**

The trials uncovered significant differences between behavior expressed by piglets castrated by tearing or cutting, indicating a higher pain level in the tearing group. It was found that the castration technique tearing increased the amount of removed tissues and might cause intraabdominal damage to the remaining tissues and vessels in a yet unknown dimension. These findings should be considered for implementation and stricter enforcement of the ban on tearing for castration.

**Supplementary Information:**

The online version contains supplementary material available at 10.1186/s40813-021-00200-7.

## Background

In pig production, castration is performed to avoid the agonistic behavior of boars and the development of boar taint, which leads to financial losses due to the meat’s inedibility. During boars’ puberty, testes start to develop the hormone androstenone, which is the main cause of the distinct boar taint. Piglet castration, i.e., the surgical removal of the testes, which is usually carried out in the piglets’ first week of life [1], is the safest and most common way to prevent the development of boar taint. To do so, the scrotum skin is usually incised with one horizontal or two vertical cuts with a scalpel [1, 2] before testes are extracted to some extent to be able to sever the spermatic cords with a scalpel or an emasculator. Another prominent way to sever the cords, however, is pulling on testes until the tissues tear off [1]. In the European Union, this technique has been forbidden since 2001 as the tearing of tissues increases pain in pigs [3]. However, it has been shown that the technique is still applied in many countries [1, 2] and even recommended [4]. This fact raises the question of whether tearing the spermatic cords is somehow advantageous, justifying the use of this method, or whether it is simply used due to lack of proper knowledge.

According to White et al. [5], pigs’ emotional or physical discomfort is difficult to measure but becomes obvious via their behavior and vocalizations. In several studies, different approaches and software have been used to assess the effects of castration, different techniques, and pain treatment [6–9]. Movements during castration have been categorized according to their intensity and duration and were found to be suitable parameters for pain response evaluation [10, 11]. Also vocalizations have frequently been used to assess pain and stress due to castration [5, 8, 10, 12]. In these and other studies, researchers made different categorizations of sounds, for example they distinguished between low-frequency and high-frequency calls with a threshold of 1000 Hz [6, 13, 14] or characterized vocalizations as ‘grunts’ (low energy), ‘squeals’ (higher energy) and ‘screams’ (highest energy) [15, 16]. In a recent trial, vocalizations have been assessed without software in a more practical approach, but only an acute onset of increased vocalizations was evaluated and no further categorization has been made [11]. Detailed reviews on the subject have recently been published [17, 18], where it was emphasized that there is no standardized procedure yet to assess the impact of castration and the efficacy of analgesic treatments. However, general findings with regard to movements and vocalizations indicate that castration induces (i) higher screams with higher frequency, energy and longer call duration [10, 13, 16] as well as (ii) increased movements of front and hind limbs and back, trembling and escape attempts [7, 19, 20].

Only a few studies have specifically discussed the advantages and disadvantages of tearing and cutting during castration. Generally, it was found that the tearing of cords takes more time than cutting [7]. It has been concluded that the most painful moment during castration is the pulling and severing of the cords [6, 21]. During tearing of the spermatic cords, the pulling force applied is more intense leading to the hypothesis that this might cause tissue damage or increase pain reaction. This might be especially relevant in anesthetized piglets due to lack of body tension. With regard to healing of the castration wound after tearing, studies have obtained different results: Bleeding was reduced [6], but the more ragged cut can complicate the healing process [22]. To the authors’ knowledge, no studies have attempted to assess anatomical integrity after castration and in how far the different castration techniques have an impact on anatomical integrity. Generally, stress is reduced during a fast procedure with minimal tissue damage [7, 23], which speaks more in favor of cutting the cords.

It was therefore the overall goal of this study to investigate differences between the ‘tearing’ and ‘cutting’ castration techniques and in how far different pain treatments affect piglets’ reactions to castration. The undertaken approach consisted of two specific aims. First, changes in vocalizations and movements during castration with the different techniques and different pain treatments were evaluated under practical conditions, for example, by maintaining realistic timing (Trial 1). With this it was aimed at determining whether it is possible to distinguish visually and acoustically between piglets’ reactions to different procedures. In a second attempt, the impact of the ‘tearing’ and ‘cutting’ technique in piglets’ physical integrity was investigated by performing castration in dead piglets to identify alterations in anatomical features due to the different procedures (Trial 2).

## Methods

### Experimental design

This study was subdivided into two experiments, which took place on different pig production farms in Germany and facilities of the University of Bonn between September 2018 and May 2019. The farms with intensive pig production had 150 (farm 1) to 500 sows (farm 2). Accordingly, different animals were used for the pain and vocalization evaluation (Trial 1, farm 1) and for the anatomical investigations (Trial 2, farm 2).

### Trial 1: Pain behavior and vocalizations

The aim of this trial was to evaluate the behavior of male piglets during castration. The behavior evaluation included the assessment of body movement during the procedure as well as the analysis of vocalizations.

### Animals, castrations and treatment groups

**Fig. 1:**
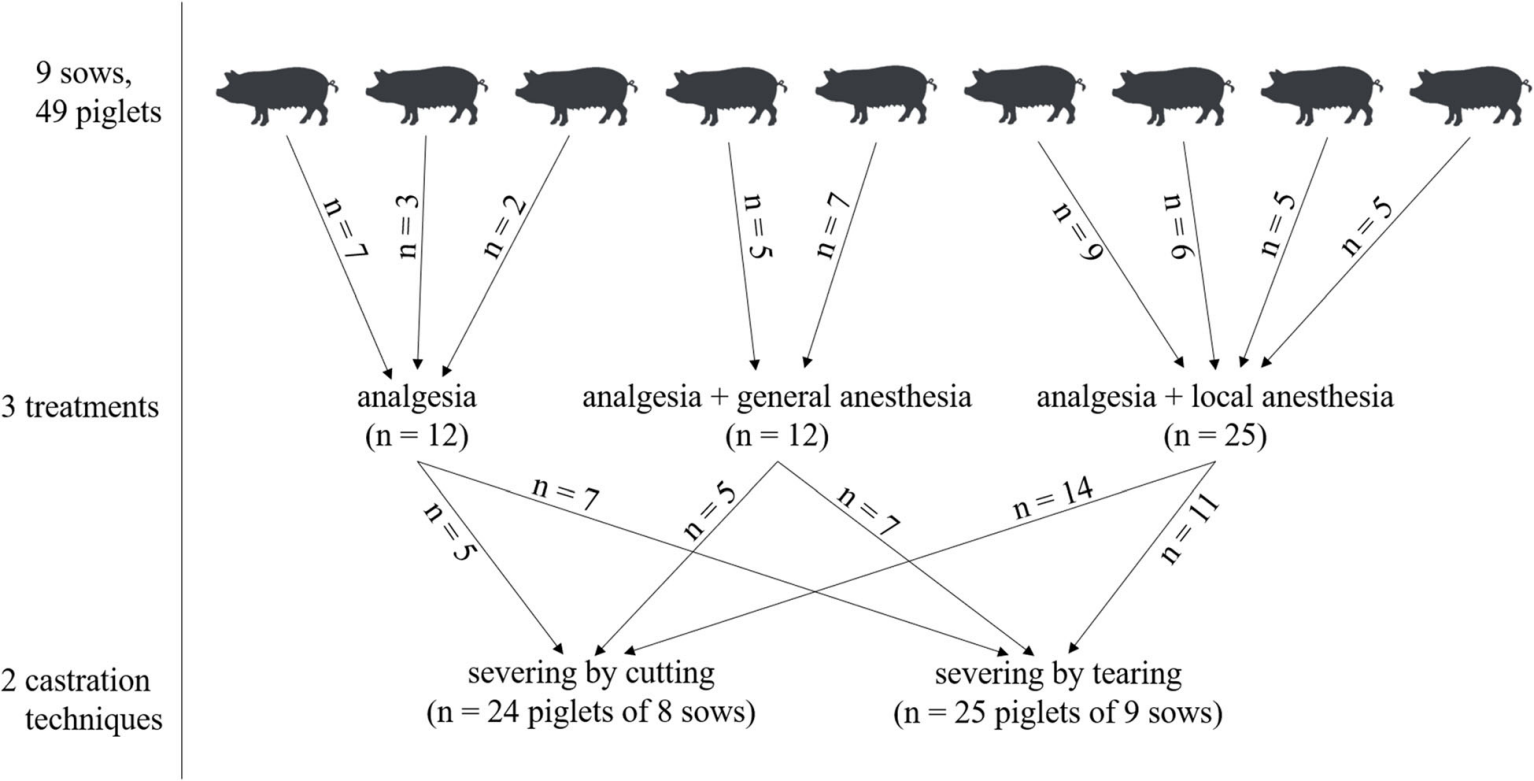
Numbers of sows and healthy male piglets allocated to the different treatment and castration groups

For this part of Trial 1, a piglet production farm (farm 1) was chosen where castration was usually performed by tearing off testes and spermatic cords. Here, sows were kept in a conventional farrowing crate system with eight farrowing crates per stable unit. Each crate measured 1.83 m × 2.40 m and had partly slatted plastic flooring. Each crate contained a piglet nest heated by an infrared lamp. Apart from chains with wooden blocks no enrichment was provided. The nine trial sows ranged in parity number from one to seven with a litter size of 14 ± 3 piglets. Male piglets of these sows (*n* = 50, 2–9 piglets per sow) were weighed and measured (crown-rump-length and chest circumference). All piglets of the same litter were then allocated to one of the three treatments. Only one treatment was allocated per litter or respectively per sow, because it was aimed at simulating the management of a litter undergoing treatment and castration under practical conditions in the superordinate project. Differences in group sizes resulted from different piglet numbers per sow (Fig. 1).

All 50 male piglets ((German Large White × German Landrace) × Duroc, 2–7 days old) were administered intramuscular analgesia (0.3 mL of meloxicam) 30 min before castration. One piglet had to be excluded from the trial due to hernia. In the first group, piglets were castrated with previous administration of analgesia only (*n* = 12). Piglets in the second group were generally anesthetized by an intramuscular injection with a mixture of ketamine and azaperone (0.3 mL/kg bodyweight) (*n* = 12). Piglets belonging to group three were locally anesthetized by two intrafunicular and two intratesticular injections of procaine (4 × 0.25 mL) (*n* = 25). Of all trial piglets, 25 (*n* = 7 (group 1), *n* = 7 (group 2), *n* = 11 (group 3)) were castrated by tearing off the spermatic cords, which was the routine procedure for castration at this farm (Fig. 1). In the remaining 24 piglets (*n* = 5 (group 1), *n* = 5 (group 2), *n* = 14 (group 3)), severing of the cords was conducted by cutting with a scalpel. All castration procedures were video recorded with sound (Cyber Shot DSC-HX100V, Sony, Tokyo, Japan) under practical conditions at castration in real time, while the camera was held by a researcher at a distance of 20–30 cm from the piglet. The camera was equipped with a stereo microphone to record realistic 5.1 channel surround sound. All castrations were performed by the same farm employee while piglets were held by a second person in the farrowing unit. After the treatments and castrations, piglets were returned to the sow. Piglets that were generally anesthetized were kept in perforated Euronorm boxes (40 × 60 × 32 cm; 0.24 m^2^) for about 4 hours until regaining full consciousness. On the day after the castrations, wounds were checked for anomalies, such as continuous bleeding, exudation, hematoma, or remains of the spermatic cords protruding from the wound, and incision lengths were measured.

### Analysis of video tapes

The duration of castration was measured from the first incision to the severing of the second testicle. However, the recording under practical conditions did not allow a numerical analysis of the frequencies. The castration procedure was subdivided into eight successive events: first incision, first extraction, first pulling, first severing, second incision, second extraction, second pulling, and second severing. For each of these events, the variables volume, kind and duration of vocalization, and kind of body movement were assessed with scores (Table 1), i.e., eight scores per parameter per piglet were recorded. Accordingly, each piglet was assigned 24 scores in total. In the following, the sum of all eight scores for each parameter was calculated.

**Table 1:** Description of scores for the assessed behavior parameters during the video analysis

Parameter	Volume of vocalization	Kind of vocalization	Kind of movement
Score			
1	No vocalization	No vocalization	No movement
2	Silent grunting	Short, rare vocalization	Minor movement
3	Low squealing or whimpering	Discontinuous vocalization with longer breaks (> 3 s)	Stretching of legs and head shaking
4	Moderately loud screaming	Discontinuous vocalization with shorter breaks (< 3 s)	Struggling and occasional escape attempts
5	Deafening screaming	Continuous vocalization	Defense movements and continuous escape attempts

All videos were watched and evaluated by the same person. Prior to this experiment, inter-observer-reliability for scoring was evaluated. For the assessment of movements, videos were watched without sound to avoid distraction by noises. Accordingly, for the assessment of vocalizations, the investigator listened to sounds only without seeing the screen.

### Trial 2: Anatomy of removed tissues and physical integrity

After conducting Trial 1, it was assumed that the applied castration techniques have a different impact on the physical integrity in piglets, which was especially expected for generally anesthetized piglets because of missing body tension. Therefore, it was the aim of Trial 2 to investigate alterations of anatomical features after the castration of dead piglets, who served as models for live but anesthetized piglets. This included the visual inspection and analysis of anatomical structures in the abdominal region and urogenital tract after dissection as well as the evaluation of testes and spermatic cords removed during castration with different techniques.

### Animals and study design

**Fig. 2:**
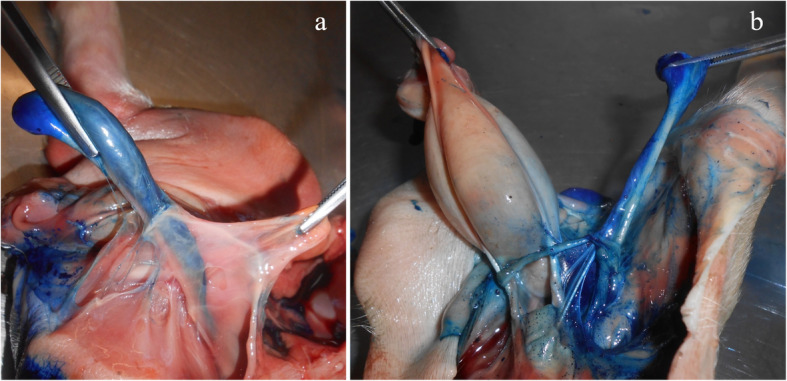
Ventral side view (**a**) and top view (**b**) of dissected piglet. The closely linked and innervated structures of the urogenital tract are outlined after dyeing testicles, spermatic cords, spermatic ducts and surrounding tissues and vessels. Testes were injected with blue ink so they appear blue in the picture. When pulling on testicle and cords, traction is transferred onto organs and tissues as shown in (**b**), possibly causing pain. Ink spreads among tissues after tearing off one testicle (**b**)

Dissections took place at the Institute of Animal Science of the University of Bonn. For this process, 28 male piglets (German Landrace × Pietrain) that naturally died between the first and fourth day of life due to undernourishment, organ failure or crushing were collected by a farmer and deep-frozen. All piglets were born and collected at the same farm (farm 2). Only piglets with intact abdomen and hind limbs were included to ensure integrity of inner structures. Before dissection of the defrosted bodies, each piglet was weighed, and the crown-rump-length as well as the chest circumference were measured. Before starting with the simulated castrations, one piglet was dissected, and the urogenital tract was exposed to gain further insight into the anatomy of the connecting tissues, ligaments and blood vessels. For this step, 1 mL of blue ink was injected into the piglet’s testicles before dissection to dye the testicles, spermatic cords and ducts (Fig. 2).

### Castrations

The castration of each piglet was performed by the same person, with the piglet body lying on its back and a second person pulling back the hind legs to create appropriate tension. The scrotum skin was opened by two vertical incisions of approximately 1 cm parallel to *raphe scroti*. After extraction of one testis, the spermatic cord was severed by cutting with a surgical disposable scalpel (Cutfix, B. Braun Aesculap AG, Tuttlingen, Germany) proximal to the testis, while the other testicle was extracted afterwards and severed by tearing. Here, the castration method was varied between the left and right testes within each piglet. To measure the power necessary for tearing off the testes, a spring scale (LTZ-1, G&G GmbH, Kaarst, Germany) was attached to the spermatic cords with a wire just proximal to the testes. This measurement was performed so that once similar measurements are conducted in live piglets, identified rupture forces can be compared to evaluate our model of dead piglets. This procedure was video-recorded for each piglet to determine the moment of rupture and read the scale indication.

### Characterization of removed tissues

**Fig. 3:**
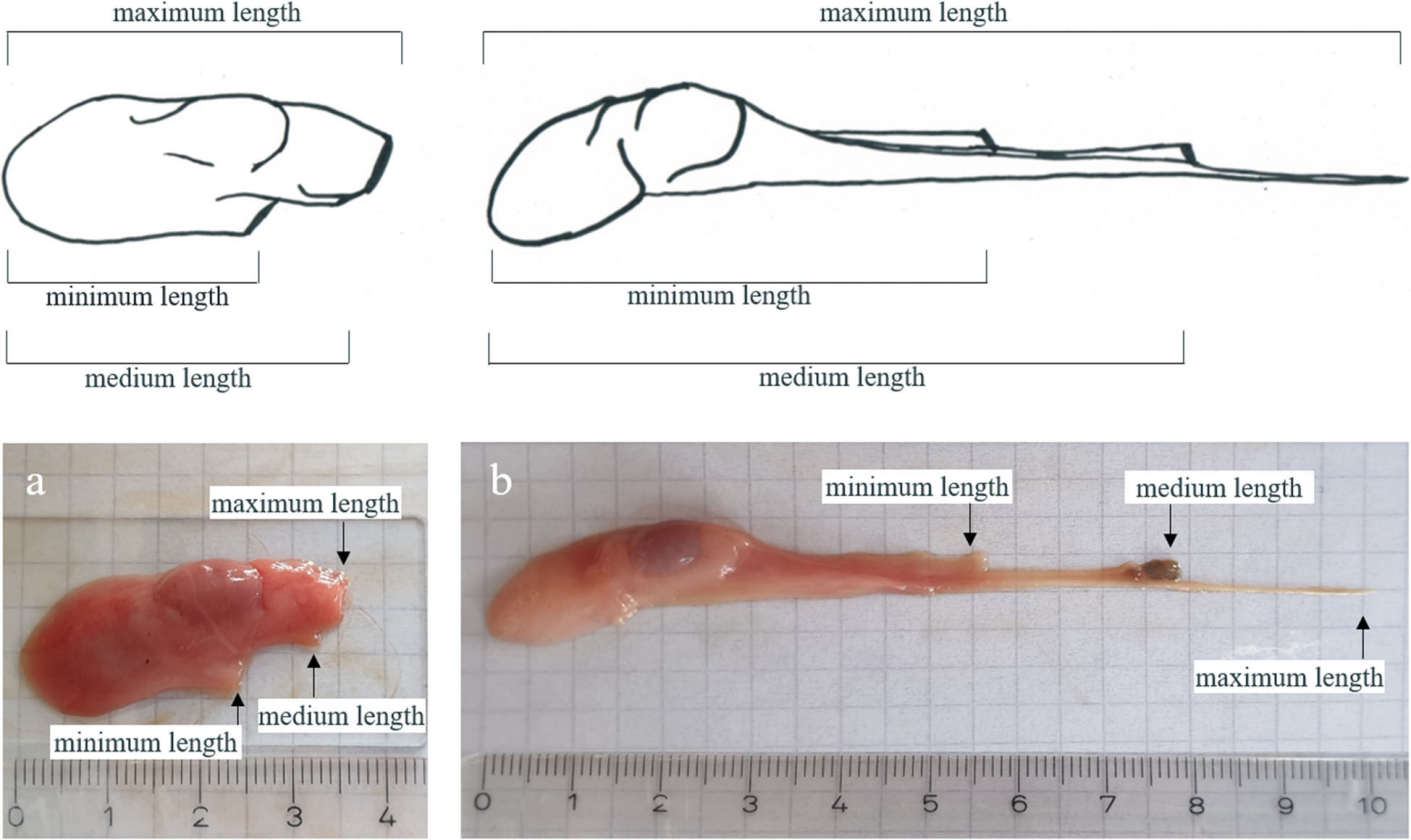
Minimum, medium and maximum lengths in cut (**a**) and torn-off (**b**) testicles

The removed tissues were collected during castration and weighed and measured afterwards. In cut-off testes, the distance from the distal end of removed tissues to the most distal cut surface (minimum length) and most proximal cut surface (maximum length) as well as to the intermediate cut surface (medium length) were measured (Fig. 3). Measurements in torn-off tissues were performed accordingly. Since differences with regard to body side or animal could not be excluded and might affect the location of cutting, the distal end carried out to be the most reliable reference point. Tissues were placed on microscope slides to closely evaluate the appearance of cut and ruptured ends using a light microscope (DM300, Leica Microsystems, Wetzlar, Germany). For this, cut surfaces were categorized as ragged or smooth and the presence of blood vessels and spermatic duct were visually inspected.

### Dissections

After removal of testes, piglets were cut open by a longitudinal cut from the cranial teats along the navel’s right side to the scrotum. The colon was removed without damaging the urogenital tract, and the bladder was carefully dissected. Remains of spermatic ducts were located in the urogenital tract proximal to the urinary bladder, secured with hemostat forceps and measured in length. In case no remains were traceable, this was noted along with any other observations regarding the castration procedure.

### Statistical analysis

Data collected during trials and video analyses were transferred to an Excel spreadsheet (Microsoft, Redmond, WA). Statistical analysis was performed with the SAS system 9.4 (SAS Institute Inc., Cary, NC) for both trials. In Trial 1, data were compared between pain treatments, castration techniques, and castration events, while for Trial 2, data between castration techniques and body sides were compared. Comparisons between these factors were performed with the Kruskal-Wallis-Test or Wilcoxon signed rank test where appropriate. Furthermore, the Spearman rank correlation procedures were used to calculate correlations between the variables of both trials. The level of significance was set at *P* < 0.05, while *P* < 0.01 was regarded as highly significant and *P* < 0.1 as a tendency. Descriptive data are presented as means ± SD. The scoring results are presented as median values.

## Results

### Descriptive statistics of animal characteristics and physiological data (Trial 1)

The mean weight of piglets in Trial 1 was 1.83 ± 0.45 kg, with a mean crown-rump-length of 39.61 ± 3.74 cm and a mean chest circumference of 27.63 ± 2.58 cm. The mean age was 4.18 ± 1.84 days and did not differ among the treatment groups. The mean incision lengths measured at the day after castration were 1.34 ± 0.26 cm (right) and 1.46 ± 0.36 cm (left). Circumference correlated positively with crown-rump-length (*r* = 0.643; *P* < 0.01), weight (*r* = 0.929; *P* < 0.01) and testicle weight (*r* = 0.309; *P* < 0.05). Crown-rump-length correlated positively with weight (*r* = 0.705; *P* < 0.01).

### Effect of treatment group (Trial 1)

**Fig. 4:**
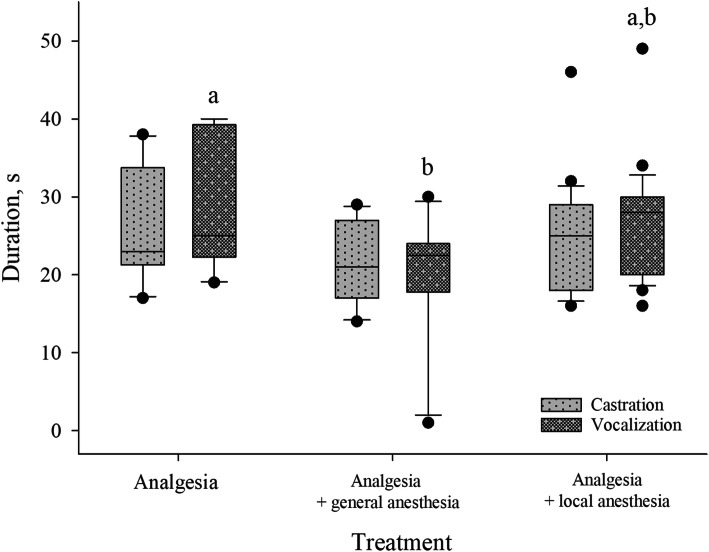
Distribution of the duration of castrations and vocalizations according to the three treatment groups. Different letters indicate significant differences between treatments (*P* < 0.05)

The average duration of the castration (i.e. first incision to second severing) was 25.94 ± 10.23 s. The different treatment groups did not significantly influence the duration of the castration procedure (Fig. 4).

The mean duration for the vocalization during castration was 27.09 ± 10.44 s. Anesthetized piglets vocalized for a shorter period of time than piglets castrated under analgesia (*P* < 0.05), while piglets receiving local anesthesia by injection did not differ from the other two groups (Fig. 4). The duration of the castration correlated positively to the duration of piglets’ vocalizations during the procedure (*r* = 0.873, *P* < 0.01).

**Fig. 5:**
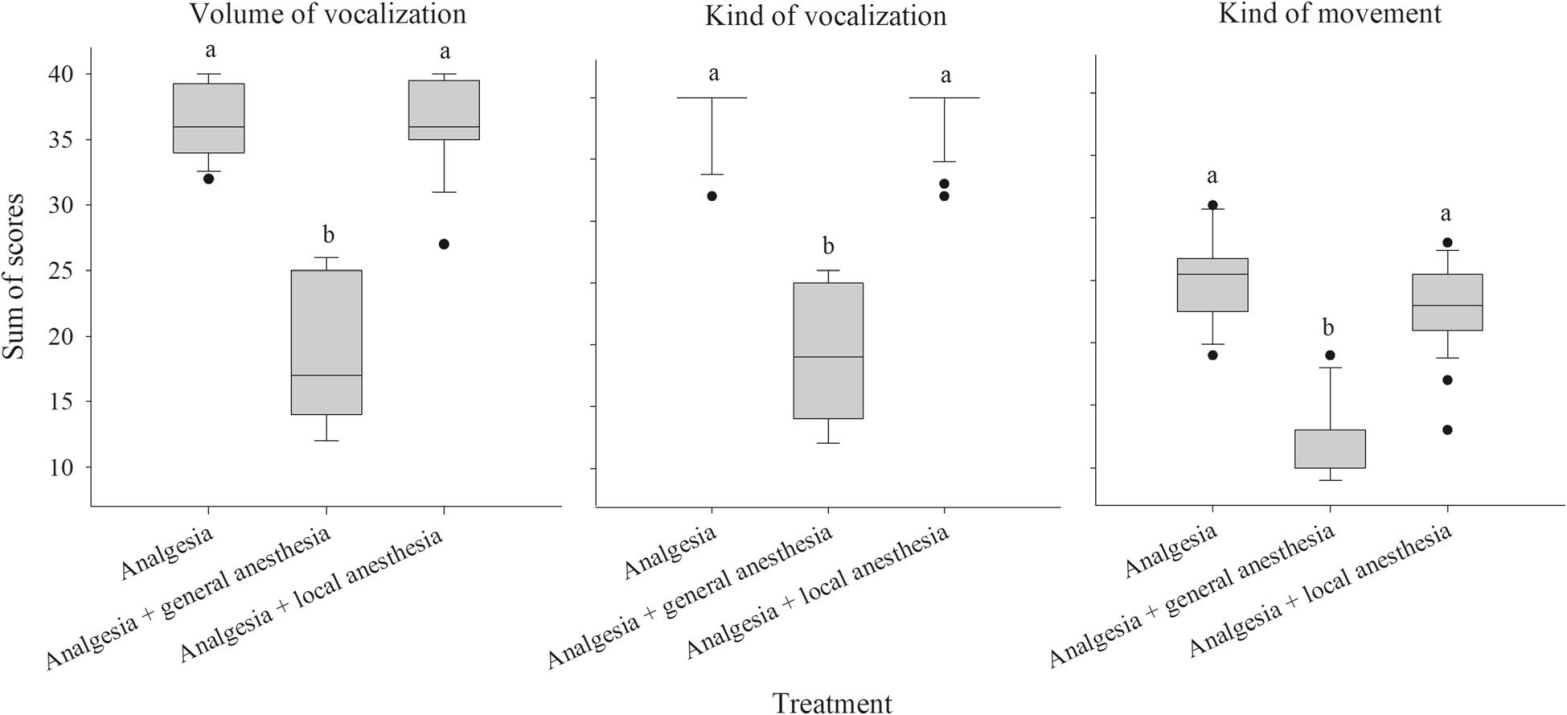
Shown are the sums of scores of the pain behavior assessment (volume of vocalization, kind of vocalization and kind of movement) according to the three treatment groups. Different letters indicate significant differences between treatments (*P* < 0.01)

**Fig. 6:**
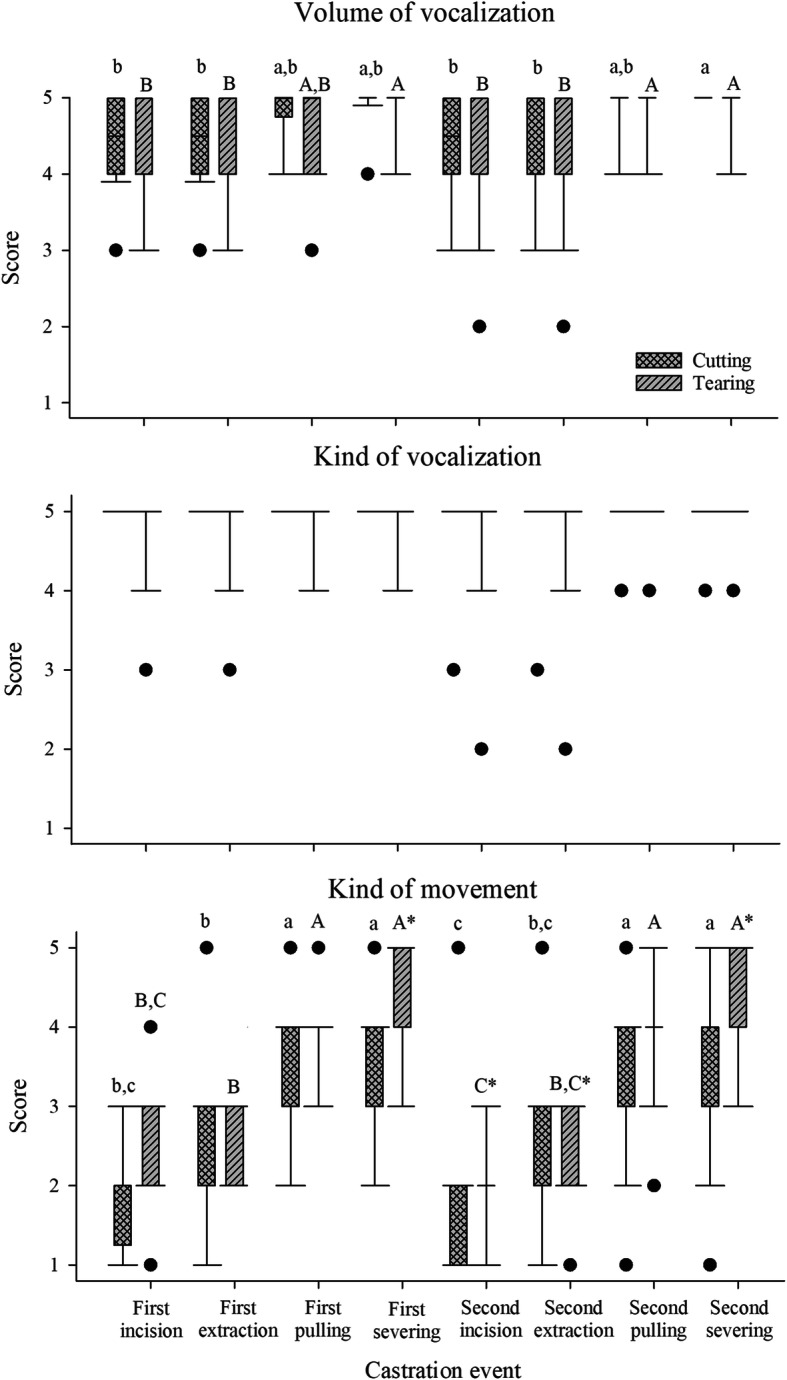
Scores for the behavior parameters volume and kind of vocalization as well as kind of movement with regard to the different castration events and castration technique groups (cutting and tearing) in a subset of data without the general anesthesia group. Different letters indicate significant differences between events (*P* < 0.01) and stars within an event between techniques (*P* < 0.01). Effect of castration technique on behavior (Trial 1)

Figure 5 presents the pain parameters volume and kind of vocalization as well as the kind of movement according to the three treatment groups. Shown are the sums calculated from the scoring of the castration events. Treatment had an effect on these variables, as the general anesthesia group obtained lower scores for the volume and kind of vocalization and kind of body movement (*P* < 0.01), but there was no difference between the other groups. According to this finding, the general anesthesia group was excluded from the comparison of behavior scores between castration techniques and events, as shown in Fig. 6.

### Effect of process event (Trial 1)

The single process events were scored to investigate possible effects on the behavior variables along the procedure. It was shown that the castration event did not influence the kind of vocalization, as piglets vocalized continuously and at a very high level at all time points (Fig. 6). However, both the volume of vocalization and the kind of movement varied significantly between the processing events (*P* < 0.01) (Fig. 6). The expression of both variables increased during the course of each testicle removal, with the incision and extraction of testicles causing lower vocalizations and fewer movements than the pulling and severing of spermatic cords (*P* < 0.01) (Fig. 6).

Castration with severing of spermatic cords by tearing with a mean of 23.64 ± 10.06 s was shorter than castration with severing by cutting with a scalpel (28.43 ± 10.03 s) (*P* < 0.01). Accordingly, the recorded vocalizations were also longer in the cutting than in the tearing group (28.91 ± 10.07 and 25.33 ± 10.70 s, respectively) (*P* < 0.05).

When comparing the effects of the different castration techniques during the single processing events, no significant differences were found for the vocalization variables (Fig. 6). The median scores attributed to the processing events were 4 and 5 for the volume and 5 for the kind of vocalization (Fig. 6). There was, however, a higher variation in the kind of movement during the different castration events for both castration techniques (*P* < 0.05): When cutting the spermatic cords, median scores of 2 (first and second incision, second extraction), 2.5 (first extraction), 3 (first pulling), 3.5 (first severing and second pulling) and 4 (second severing) were observed, while during tearing of the cords, median scores of 2 (first and second incision, second extraction), 3 (first extraction) and 4 (first and second pulling, first and second severing) were obtained. For the removal of the first testis, the only difference in the kind of movement between piglets castrated by tearing and those castrated by cutting was observed during the severing (*P* < 0.01), were found (Fig. 6). When focusing on the second removal, a significant difference in movements was observed for the incision (*P* < 0.01), extraction (*P* < 0.05) and severing (*P* < 0.01), with more intense movements when spermatic cords were severed by tearing. Additionally, on the day after castration, there was a trend for remains of spermatic cords protruding from castration wounds more often after severing by tearing (24.00%) than after cutting (4.35%) (*P* < 0.09), but there was no difference with regard to other parameters such as bleeding which occurred after tearing (16.00%) and after cutting (30.43%).

### Descriptive statistics of animal characteristics and anatomy of removed tissues (Trial 2)

The mean piglet weight was 1.34 ± 0.32 kg, while the mean crown-rump-length and chest circumference were 37.76 ± 2.40 cm and 23.74 ± 2.12 cm, respectively. Body weight correlated positively with crown-rump-length (*r* = 0.818; *P* < 0.01) and chest circumference (*r* = 0.969; *P* < 0.01), and crown-rump-length and chest girth also correlated (*r* = 0.826; *P* < 0.01). Furthermore, testes weight showed moderate positive correlations to body weight (*r* = 0.657, *P* < 0.01) and crown-rump-length (*r* = 0.344, *P* < 0.05). The mean force required to tear off the spermatic cords was 8.63 ± 1.58 Newton (N) with a range of 6.00 N to 11.00 N. The weight of severed testicles and the relative testicle weight correlated moderately with the force applied during tearing (*r* = 0.546 and *r* = 0.464, respectively, *P* < 0.05).

### Effect of castration technique on anatomy (Trial 2)

**Fig. 7:**
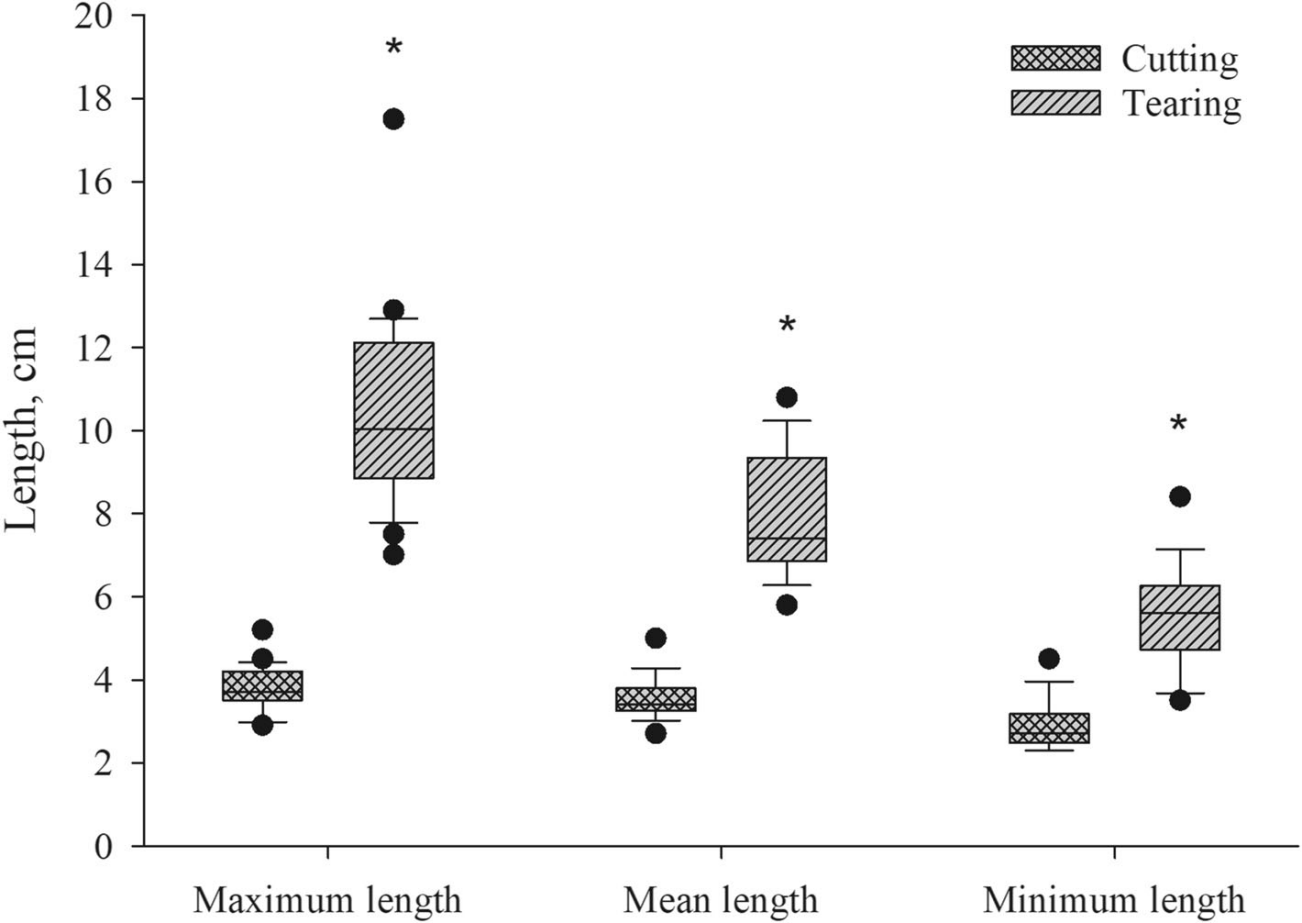
Length of testicles and spermatic cords after severing by tearing or cutting. Stars indicate significant differences between techniques (*P* < 0.01)

The minimum, mean and maximum lengths of the testicles and spermatic cords were extended when severing was realized by tearing (*P* < 0.01) (Fig. 7). The lengths of the spermatic cord remains in situ were not different, with mean lengths of 4.18 ± 1.13 cm after cutting and 4.13 ± 3.44 cm after tearing. Remains of the spermatic cords protruded more often from the incision after tearing off the tissues (19.23%), but not after cutting (0.00%) (*P* < 0.05). Furthermore, absolute weight of the severed testes and spermatic cords was not significantly different between the two groups.

The microscopic evaluation of spermatic cords and ducts showed that cut tissues had an even cut, while torn tissues showed ragged separation areas. The torn spermatic ducts appeared to be thinner in diameter. Furthermore, it was visible that in cut tissues, structures such as blood vessels and the spermatic duct were still attached to the spermatic cord, while in torn tissues, these structures were separated from the spermatic cord and generally had a more tattered appearance.

The maximum testicle length strongly correlated to the mean and minimum testicle lengths (*r* ≥ 0.843, *P* < 0.01), which also showed a positive correlation (*r* = 0.784, *P* < 0.01). Weak correlations were found between testicle length and testicle weight (0.346 ≤ *r* ≥ 0.420, *P* < 0.05). The length of the spermatic duct remains in situ correlated to piglet weight in piglets castrated by cutting (*r* = 0.513, *P* < 0.05).

**Fig. 8:**
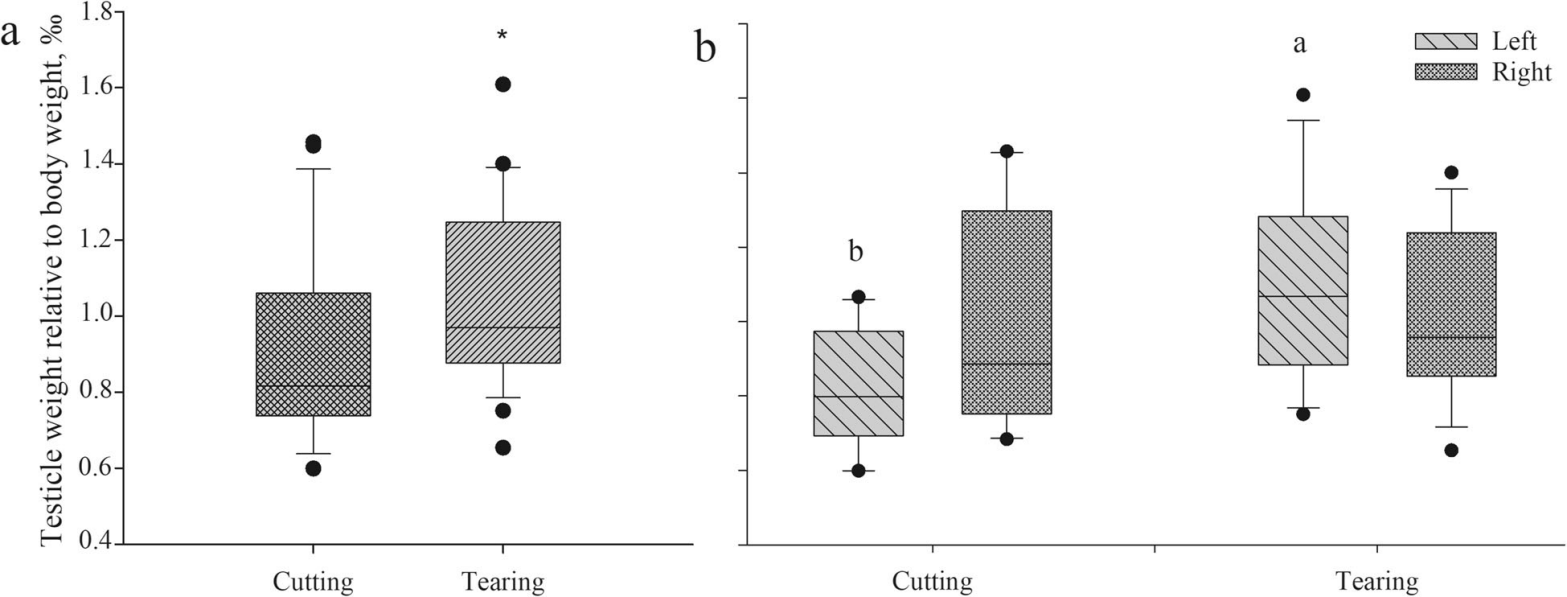
Distribution of testicle weight relative to body weight (per thousand) for the different castration techniques tearing and cutting (**a**) and relative testis weight (per thousand) according to body side and castration technique (**b**). Stars indicate differences between techniques (*P* < 0.05) while different letters indicate significant differences between techniques within a side (*P* < 0.01)

Figure 8 represents the distribution of testicle weight relative to body weight for piglets castrated by tearing and cutting. The mean relative testicle weight of 1.05‰ in piglets castrated by tearing was larger than that in piglets castrated by cutting (0.91‰) (*P* < 0.05) (Fig. 8a).

### Effect of body side (Trial 2)

When including the effect of left and right testicles in the analysis, similar results were found. The comparison of minimum, mean and maximum lengths of torn and cut testicles of both body sides revealed higher values in the torn-off tissues (*P* < 0.01). With regard to testicle weight, the values were higher in torn left tissues than in cut left tissues (*P* < 0.01). This difference was not observed when comparing torn and cut tissues on the right side.

There was no body side effect on the length of the remains in situ. Regarding the relative testis weight (per thousand), again a difference was found for the left testis: the mean weight after tearing left testicles (1.09‰) was relatively heavier than that after cutting left testicles (0.81‰) (*P* < 0.01). However, there was no difference at the right-hand side (Fig. 8). Relative testis weight correlated positively to maximum testicle length (*r* = 0.358, *P* < 0.05) and negatively to the length of the spermatic duct remains in situ (*r* = − 0.383, *P* < 0.05).

## Discussion

One aim of the present study was to determine whether it is possible to detect indicators for pain and differentiate between reactions to certain procedures with the human eyes and ears under practical conditions (Trial 1). Castrations of anesthetized piglets increase due to legal obligations to improve animal welfare. This means that especially generally anesthetized piglets show ideally no reactions to castration, however, other aspects such as the least impeding castration technique have to be considered. Indeed, the behavior of generally anesthetized piglets differed clearly from locally anesthetized and non-anesthetized piglets. Additionally, the expressed body movements of piglets castrated by tearing were more intense than those of piglets whose spermatic cords were severed by cutting with a scalpel. Interestingly, results of Trial 2 also indicate alterations in anatomical structures and an increased removal of tissue, when testes are torn off. This indicates a higher risk for anatomical impairment during castration in case of lack of body tension.

### Effects of castration technique and event on vocalizations and movements (Trial 1)

Categorization of vocalizations as grunting, squealing and screaming has been mentioned previously [15, 16]. For this, sounds were analyzed with a signal processing system and characterized according to the amount of energy detected and other sound parameters [15]. For the vocalization assessment in the present study, sounds were not converted and analyzed via software, because it was aimed at conducting an auditory approach under largely practical conditions. Therefore, it was not possible to numerically determine sound parameters such as entropy, energy or frequency and the generated data is not fully comparable to other study results. Similarly, Abendschön et al. [11] applied a more practical approach by determining an acute onset of vocalization by human hearing. They, however, did not further categorize the perceived sounds. We developed a score based on the classification previously applied [15, 16] but we observed that there are different types of screams. We therefore differentiated between ‘moderately loud screaming’, and the even more intense ‘deafening screaming’. The latter scream type can be described as an extreme quavering sound similar to piglet screams elicited during crushing events. As piglets emitted these different kinds of screams during castration, this categorization and the applied scoring method were found to be more suitable for the appropriate identification of pain-related vocalizations. Furthermore, it has been found previously that more specified scoring scales are more accurate than scores with fewer parameters [24]. For the same reason, the whole castration process was subdivided into eight consecutive events to be able to perform a more detailed evaluation.

With regard to the castration events, volume of vocalization and kind of movement revealed significant differences. This result supports the finding from Keita et al. [19], who claimed that movement of front and hind legs, trembling, and other body movements can depict the effect of castration. In the present study, movements were more intense during the pulling and severing of testicles, which confirms earlier findings [6, 21]. Scoring of the castration procedure did not reveal differences between the behavior of pigs castrated by tearing and those castrated by cutting. Both castration techniques evoked strong signs of defense behavior and struggling, which might not have been easily distinguishable for the human eye and ear. In previous studies, it was also not possible to define differences between tearing and cutting based on vocalizations [6, 7]. Taylor and Weary [6] assumed that vocalizations can reach a maximal level caused by a maximal pain reaction and concluded that the pain caused by these castration techniques is expressed in an equal manner, which makes it difficult to detect differences, as confirmed by Marchant-Forde et al. [7]. However, when focusing on single castration events, it was possible to detect differences in the volume of vocalization and the kind of movement during severing when testicles were torn off. This observation indicates that the tearing was more painful, especially after the first testicle had already been torn off. It is assumed that this difference is caused by an accumulation of pain and stress and a repeated traction on the abdominal cavity and urogenital tract. Similar observations have been made after performing several procedures consecutively in piglets [23, 25]. Our results confirm previous studies [10, 11], indicating that particularly the kind of body movement can be considered a reliable indicator for the assessment of pain and distress during the castration of piglets, as it is able to determine differences between different castration techniques and pain treatments.

Both the duration of vocalization and the duration of the castration procedure were shorter in piglets castrated by tearing. This finding contradicts results from Marchant-Forde et al. [7]: The severing of spermatic cords by tearing took almost half a minute longer than severing with a scalpel, which was explained by the time needed to carefully take hold of the spermatic cords to ensure a steady pulling. However, as in the present experiment, the castration was performed by an employee who routinely uses the tearing technique, and he did not pay special attention to its execution. Then again, the performing employee was not as trained in cutting the spermatic cords with a scalpel as he was in tearing, which might have prolonged the cutting procedure and caused the differences. During another trial, it took an experienced stockperson approximately 10 seconds only to castrate piglets by cutting the spermatic cords [26]. Although it is preferable to shorten the procedure to reduce the stress that piglets are exposed to, it is assumed that tearing does not generally take less time, depending on the training of the stockperson.

### Effects of anesthesia treatment on vocalizations and movements (Trial 1)

The castration of piglets induces intra- and postoperative stress and pain [5, 23, 27]. From this year on, piglets in Germany have to be surgically castrated under general anesthesia induced by inhalation or injection [2]. Options for mitigating pain during castration have been discussed in detail [17, 18]. Also in the present study, it was analyzed whether treatment with general or local anesthesia affected the physical reactions of piglets during castration. The control group here was the group of piglets receiving analgesia only, which is legally required in Germany. The duration of the castration procedure did not differ among the groups. It was expected that the castration would take less time in the general anesthesia group, as these piglets were unable to act out with defensive behavior. This expectation was only confirmed by a numerical difference between the treatments. However, it was reported by the castrating stockperson that handling and castration of anesthetized piglets was not always easy, as loose limbs had to be secured to avoid interference with surgical tools.

The results of the behavior assessment via scores revealed that the only treatment with significantly lower body movements and vocalizations was the general anesthesia treatment. This finding was expected, as the piglets were unconscious and therefore showed little movement and few vocalizations and served as a control for the applied methodology. Sutherland et al. [9] found similar results. Nevertheless, although piglets received the recommended drug amount according to their weight in the present study, it can be assumed that they experienced some distress, as anesthetized piglets also vocalized to some extent. Given that no differences were found between the control and local anesthesia groups, it might be suspected that these treatments were less effective in reducing pain, confirming previous findings [28] but contradicting others [5, 10, 27].

In Trial 1 of this study, piglet behavior was scored during castrations in the farrowing unit. Piglets were not transferred to a separate room, as has been done in earlier studies [7, 14], to ensure practical conditions and realistic procedure times and avoid an alteration of behavior due to additional stress. The recording in scores of vocalizations and movements during procedures seems to be the only appropriate method under practical conditions, as sounds of other pigs and technical equipment in the farrowing unit would distort frequency analyses. One limitation of the present study is that treatments were allocated per litter, so that a potential sow effect cannot be excluded for treatments. Additionally, when interpreting the scoring results, it has to be considered that vocalizations are subject to individual animal differences, making interpretation more difficult [28, 29]. In the present study, it was observed that tight fixation limited movements, especially in smaller piglets, and could therefore lead to lower scoring. This observation was confirmed by weak, but significant correlations between weight, circumference and movement score. Additionally, when evaluating pain with scores for which intra- and inter-assessor differences are known [30], assessors should be experienced and should be the same person. More objective evaluations, e.g., analysis via software [30], would be preferable but are hardly feasible under field conditions.

### Effects of castration technique on anatomical integrity (Trial 2)

Even if analgesia and/or anesthesia are applied for the mitigation of pain, the least detrimental castration technique should be chosen to avoid any redundant distress and pain [3, 6, 31]. However, in several European nations and other countries with commercially important pig production, the tearing technique is still routinely applied [1, 2, 4]. To the authors’ knowledge, this is the first experiment to investigate the effects of different castration techniques in situ. The goal of Trial 2 was to determine whether the tearing technique induces more tissue damage than the cutting technique. Here, piglet bodies served as a model for live piglets, and the findings cannot readily be transferred to live animals. Nonetheless, it is assumed that results are to some extent applicable to the castration of generally anesthetized piglets due to the lack of body tension. However, other factors, such as blood irrigation or tissue integrity might affect the applicability of the present observations, which have to be interpreted with care. It has been claimed that the advantage of pulling the spermatic cords until they tear off is the reduced bleeding [6]. This claim can supposedly be traced back to the fact that pulling induces a recoiling of *A. testicularis* and lumen constriction [32]. In human medicine, the tearing of tissues is applied, for example during Caesarian section [33], since it is assumed to be more indulgent as it decreases blood loss and can be performed faster [34, 35]. In this case, however, tearing is not used to remove organs, as is the case during piglet castration. Tearing off the spermatic cords might not reduce bleeding but is perceived as such, as the tearing point might be more proximal, and the bleeding is therefore less visible [6]. Bleeding was not examined in the present study. According to the results of this experiment, there was no difference between the spermatic ducts in situ after tearing or cutting.

However, it was observed that the remains of the torn spermatic ducts were thinner, as they were stretched during the pulling. Furthermore, the mean length of the removed testicles’ blood vessels was significantly longer in torn tissues than in cut testicles. It can therefore be assumed that the tearing point was more proximal than the cutting point, which supports the assumption of Taylor and Weary [6]. This result should be kept in mind when considering local anesthesia for reducing pain during castration, especially when injection is performed intratesticularly and not intrafunicularly, since the pain might occur more proximal to the injection location of the local anesthetics, as was claimed by Thornton and Waterman-Pearson [36]. This assumption is supported by a study showing no differences in cortisol levels after tearing off spermatic cords in locally anesthetized and non-anesthetized calves [29].

In addition, it was discovered that the amount of removed tissue is also dependent on the size of the piglet. Significant differences were found in the weight and relative weight of removed tissues after tearing and cutting. Moreover, weight was significantly higher in tissues severed by tearing, indicating that more tissue was removed when using this technique. However, this was only the case for the left testicles; on the right side of the body, the castration technique had no effect on testicle weight. The reason for this discrepancy is not clear. An explanation could be the naturally occurring asymmetry in weight, size or position of paired organs such as kidneys [37, 38] and testes [39–41]. Another reason could be that the simulated castrations were performed by a right-handed person, and the impact might have been different on the left and right testicles. However, this difference between the left and right sides was not found during a pretest in live animals (*n* = 8), in which one testicle was severed by tearing and the other by cutting, because here, the difference in the weight of the removed testes was significant for cut and torn left testicles as well as for cut and torn right testicles. In the course of this pretest, it was observed that in all cut testicles, the surrounding connective tissue was present, while it could only be found in some torn testicles [42]. It was assumed that a certain grip during the severing resulted in the sliding off of connective tissues, which then remained in the scrotum. This situation led to a wide range of weights in the torn testicles and might have also influenced the weights in the present experiment [42]. In the present study, testis weight correlated significantly to the length of removed testicle, which was significantly longer in torn testicles. This result indicates that more tissue was removed when tearing the tissues. As it is recommended to remove as little tissue as possible [43], the unnecessary removal of further tissue might have negative effects.

During castration, strong traction is applied on piglets’ testicles, triggering nociceptor impulses [29]. Testicles are highly innervated by sympathetic nerve fibers and nociceptors, which are able to perceive strong pain [44]. Since testicles develop abdominally and descend to the scrotum shortly before the time of birth [45], a strong interconnection between testicles and abdomen remains postpartum. Testicles are supplied by abdominal blood vessels and neuroplexus, for example, *A. testicularis* arises from *A. abdominalis* [46]. These complex innervated structures have been described before, indicating a strong pain perception [39]. For better visualization of the link between abdomen and testes, an uncastrated piglet was dissected in the course of this experiment, and while pulling on the testicles, the interaction of the whole abdominal cavity became visible. According to Taylor and Weary [6], this traction does not directly imply a damaging of tissues, but is likely to cause pain felt up into the inguinal canal. Apparently, the pain is especially intense if the *Tunica vaginalis* is not removed from the testicles before pulling due to the impact on the inguinal canal [6].

Part of this experiment was to define the force necessary for severing the spermatic cords in dead piglets. Here, a mean force of 8.63 N had to be applied to tear off the cords, which is equivalent to a mass of 0.88 kg. Woollard and Arnold Carmichael [47] placed weights on anesthetized human testicles and found that a mass of 0.3 kg (2.94 N) induced discomfort, while a mass of 0.6 kg (5.98 N) caused pain. Strong pain was further evoked by a mass of 1 kg (9.81 N) [47]; therefore, it can be assumed that the force of 8.63 N in the present experiment might cause strong intra-abdominal pain in live piglets, which counteract the pulling with their body tension. The pulling on spermatic cords evokes a damaging of the structures remaining in the body, possibly perceived as poorly locatable intra-abdominal discomfort [36]. Therefore, Stafford et al. [29] also assumed that severing the spermatic cords by cutting is a less traumatic procedure than tearing. In a previous trial, when severing was performed by tearing, it was found that testicle weight was higher in anesthetized piglets than in conscious piglets [26]. It was assumed that this effect was caused by the lack of body tension in anesthetized piglets. The reduced body tension occurs due to the decreased muscle tone during anesthesia [48]. However, the hypothesis that testis weight is higher in anesthetized piglets after tearing the spermatic cords could not be proven in Trial 1 and might require a larger test population.

Although wound healing was not specifically addressed in this study and therefore not monitored for a longer period of time, it was observed in several animals immediately after castration (Trial 2) and on the day after (Trial 1) that the remains of spermatic cords hung out of the castration wound after severing the spermatic cords by tearing. This finding confirms observations from previous castration studies [28, 49, 50]. In the days after castration, tissues protruding from the wound are disadvantageous, as they impair closure, can serve as a site of entry for pathogens and therefore increase the risk of wound infections [28]. In future studies, castration wounds should be reinvestigated after several days to evaluate differences during healing. Stafford et al. [29] found no differences with regard to the duration of wound healing after castration by tearing and cutting in calves. However, it is not known how fast intra-abdominal injuries caused by tearing off the spermatic cords heal [51].

## Conclusions

In the course of this experiment it was possible to detect differences in behavior expressed by anesthetized and non-anesthetized piglets, indicating that the applied general anesthesia was successful in mitigating pain perception. An assessment of pain is possible under practical conditions without the use of sound analyses. Some behavioral differences were found between piglets castrated by tearing or cutting and physical integrity was altered by the different techniques. By means of anatomical characteristics, it was found that the tearing castration technique increases the amount of removed tissues and might cause damage to the remaining tissues and vessels in a yet unknown dimension. This should be further investigated in future studies and considered for implementation and stricter enforcement of the ban on tearing for castration. For castration, pain mitigation should be applied and the technique with the least pain-inducing potential should be chosen, which is, according to the results of this study, the cutting of spermatic cords.

## Supplementary Information

**Additional file 1.**

## Data Availability

The datasets used and/or analysed during the current study are available as supplementary information.
